# Co-development of a protocol for a randomised controlled feasibility trial of CIRCuiTS™ cognitive remediation therapy for people with multiple sclerosis

**DOI:** 10.1186/s40814-026-01862-2

**Published:** 2026-06-20

**Authors:** Rebecca J. Thomas, Matteo Cella, Peter A. Brex, Eli Silber, Kate Fifield, Patrick Burke, Mary Summers, Sara Simblett, Til Wykes

**Affiliations:** 1 Department of Psychology, Institute of Psychiatry, Psychology and Neuroscience, King’s College London, London, UK; 2 South London and Maudsley NHS Foundation Trust, London, UK; 3 Department of Neurology, King’s College Hospital NHS Foundation Trust, London, UK; 4 CIRCuiTS MS Steering Committee, King’s College London, London, UK; 5 Blackheath Brain Injury Rehabilitation Centre, London, UK

## Abstract

**Background:**

Cognitive difficulties are common in people with multiple sclerosis (MS) and predict poorer quality of life, yet effective treatment is lacking. Cognitive remediation therapy (CRT) improves functioning in individuals with severe mental health conditions and could benefit people with MS if adequately adapted. Recognising patient and public involvement (PPI) benefits in developing acceptable interventions, this study engaged people with MS to develop a protocol to assess the feasibility of delivering a computerised, therapist-assisted CRT programme (CIRCuiTS™) to people with MS. This paper describes this process and presents the trial protocol, highlighting how PPI informed the study design and treatment implementation.

**Methods:**

Protocol co-development involved three phases of consultation with people with MS. First, the PPI lead, who has MS, contributed along with mental health professionals to the design draft. Second, co-development using an observational qualitative design was conducted with people with MS in a structured 2-day workshop. Workshop discussions were recorded, transcribed, and analysed thematically to arrive at 24 specific, actionable recommendations. These recommendations, alongside statistical input, guided the trial design. Finally, a separate PPI group provided feedback on the trial documents.

The proposed trial will recruit 24 people with MS experiencing cognitive difficulties from a UK NHS service, randomly assigning them to receive a 12-week CIRCuiTS™ MS programme immediately or after a 13-week wait. This relatively short waiting period was recommended as likely to maintain engagement. Standard CIRCuiTS™ delivery will be adapted to MS needs by offering remote sessions, dexterity tailoring and MS-specific therapist training. Primary outcomes are feasibility and acceptability. Guided by PPI-anticipated benefits, the secondary outcomes will be goal attainment, cognition, fatigue, mood, and daily functioning. Two people with MS continue to guide the study in the oversight group and a PPI Advisory group will provide ongoing advice on trial management.

**Discussion:**

PPI, as recommended in the new CONSORT 2025 statement, strengthened the feasibility and acceptability of the trial by addressing challenges and shaping the protocol. This collaboration enhances the evaluation of CRT for people with MS and provides a model for transparent PPI reporting in trial development.

**Trial registration:**

ClinicalTrials.gov ID NCT06877273, 14th March 2025.

**Supplementary Information:**

The online version contains supplementary material available at https://doi.org/10.1186/s40814-026-01862-2.

## Background

Cognitive difficulties are common in multiple sclerosis (MS), with a pooled prevalence of 32.5% [1], and they predict poorer quality of life [2]. Impairments are most common in information processing speed and working memory and also occur in executive function, learning, memory, language and spatial processing [3]. These deficits can have significant real-world consequences. They are associated with reduced employment [4], and both quantitative and qualitative studies have highlighted their impact on everyday activities [5–7]. In the absence of safe and effective cognitive enhancement medications, a recent review concluded that cognitive rehabilitation is currently the most promising approach to address these problems [8]. Most studies, though, either concentrate on one cognitive domain, with improvements in that domain but not on real-life functioning, and others have a more general focus and show benefit on psychological symptoms but not cognition. For example, RehaCom software demonstrates a moderate–large effect on processing speed [9], but this change is in clinical test score (e.g. [10, 11]) rather than client-reported functional outcomes. Although cognitive training programmes are available, they are not widely implemented for people with MS. The lack of evidence for the effects on real-life functioning will probably continue to mean that widespread availability will be elusive.

Cognitive remediation (CR) is a specific behavioural intervention based on learning principles. It differs from many of the cognitive rehabilitation programmes previously tested in MS as it does not only depend on task practice to build cognitive resilience. CR interventions are defined as having four good practice ingredients: an active and trained therapist, cognitive exercises, teaching strategies and support to transfer gains to real life tasks [12]. It has been demonstrated to improve goal achievement and functioning in more than 130 studies in serious mental health conditions [13]. It is cost-effective and when all the ingredients are present, there is a significant boost in effect sizes [13, 14]. Unassisted digital rehabilitation interventions have shown limited and inconsistent effects in MS [15]. As a therapist-supported intervention, CR therefore has the potential to deliver meaningful and sustained benefits for people with MS, with the therapist ensuring adherence, promoting meaningful engagement, and enhancing outcomes as in other conditions [16].

To date, most effectiveness evidence supporting the CR approach is for people with mental health conditions, including schizophrenia, depression and bipolar disorder (e.g. [13, 17]). Given the similarities in cognitive difficulties in people with MS (for example, impairment of processing speed and working memory [18, 19]), CR holds promise for delivering similar benefits. Furthermore, cognitive impairment in MS frequently co-occurs with fatigue and depression, with evidence that these symptoms interact and account for significant variance in cognitive performance [20, 21]. CR’s emphasis on metacognitive strategy use may help individuals recognise and manage the impact of these factors on their thinking, and its demonstrated benefits in depression [22] further support its potential relevance for this population. However, the distinct features of MS, such as physical and sensory symptoms that can fluctuate and progress, may make CR more challenging and affect its feasibility and acceptability.

This paper describes the development of the protocol for a randomised feasibility trial to determine whether cognitive remediation adapted for people with MS is likely to have potential to improve thinking skills. The chosen software, Computerised Interactive Remediation of Cognition and Thinking Skills (CIRCuiTS™; https://www.circuitstherapyinfo.com/), contains all the four ingredients defined for effective cognitive remediation, and there is ample independent evidence of its benefits in serious mental health conditions including schizophrenia, early psychosis and bipolar disorder [17, 23–25]. CIRCuiTS™ is a web-based computerised CR programme in which a therapist guides the client through a range of cognitive exercises. It specifically targets metacognition, helping clients to develop effective problem-solving strategies to overcome real-life cognitive difficulties and produce long-lasting change. Its focus on remediating deficits in areas such as memory and executive function as well as an emphasis on improving real-life functioning suggest that it may meaningfully benefit people with MS. Additionally, it can be delivered remotely [26, 27], suggesting potential feasibility for people with MS with mobility difficulties, and can be culturally adapted [28] to increase access.

Collaboration with service users is helpful in developing feasible and acceptable studies and interventions [29]. We therefore adopted a co-development approach with people with MS to ensure that CIRCuiTS™ delivery and the trial procedures align with the MS population’s needs and priorities. This paper describes this co-development process and the resulting protocol, which will explore the feasibility and acceptability of MS-adapted CIRCuiTS™ and estimate its potential for further investigation in a larger efficacy trial. Given the increased focus on lived experience involvement in research, this work also serves as an early example of how the new CONSORT guidance on reporting patient and public involvement (PPI) [30] can be put into practice. It offers a transparent, practical model for integrating and reporting meaningful PPI in the early stages of clinical research that may be relevant to researchers developing trials in MS and other conditions.

## Methods

The methods are divided into two parts: (a) co-development of the protocol and (b) the finalised protocol incorporating the changes suggested.

### Protocol co-development process

Protocol co-development involved three phases of collaboration with people with MS:

1. The study’s PPI lead, who has MS and extensive experience of PPI in studies (e.g. [31]), advised on an initial draft protocol produced by researchers and clinicians with continuing comments via PPI representatives.
2. The PPI lead guided the design of a PPI workshop for further protocol co-development delivered online to reduce the need to travel. As fatigue is often a problem, it was agreed to divide the tasks between two consecutive days. The workshop considered the perceived benefits from CIRCuiTS™, aspects of study design and adaptations to the intervention and its implementation.
3. Following the workshop, feedback on the study documents was provided by a further PPI group with experience in research to further ensure feasibility and comprehensibility.

#### Workshop organisation

The workshop protocol was accepted by King’s College London’s Research Ethics Office (Reference Number: LRM-24/25–45,431). Participants were recruited through the PPI lead’s personal MS lived-experience blog. Participants were included if they were over 18 years old, reported being in a stable phase of their condition, were judged on interview to be able to participate meaningfully in an online workshop, and could provide a doctor’s letter confirming their diagnosis of MS. Four out of six people who volunteered provided consent and attended both workshops, receiving £300 in total as compensation for their time.

Discussions were facilitated by two researchers. The first day started with a demonstration of CIRCuiTS™, after which focus group members gave their initial impressions. The group then discussed experiences of thinking difficulties in MS and perceived barriers to and facilitators of recruitment to a trial of the programme. Day two was devoted to discussing the modifications needed to CIRCuiTS™ content and delivery to make it suitable for people with MS. Topics included the suitability of the proposed session schedule and standard intervention content, feasibility of different therapy venues, and practical challenges that might limit engagement with CIRCuiTS™.

#### Qualitative analysis

Group discussions were captured using automatic transcripts and manually corrected. Data were analysed using an adapted Framework Analysis approach, integrating both deductive and inductive analytic approaches [32, 33]. Domains identified in the Participatory Action Research model [34] as important during the design stage of a research trial informed an initial set of organisational categories for coding: PPI, recruitment strategy, intervention content, intervention delivery, therapist recruitment and training content, data collection, ethical considerations, and sustainability.

Following familiarisation with the transcripts, two researchers collaboratively conducted inductive coding within each domain using NVivo version 14 [35]. Data-driven codes were iteratively compared and grouped into higher-order themes, with the analytical framework evolving dynamically as analysis progressed. Once consensus on the hierarchical framework was achieved, an independent researcher applied the final coding framework to the transcripts to assess consistency in coding. Codes were subsequently synthesised into broader themes, which were reviewed and refined for coherence and distinctiveness. Inter-rater agreement was calculated at the code level using Cohen’s Kappa (0.78), indicating substantial agreement. Any disagreements were discussed and resolved to reach consensus. The final coding framework is presented in Table S1 (Additional File 1), and the analysis informed the actionable recommendations shown in Table 1.

**Table 1:** Actionable recommendations from co-development workshops

PPI advisors to the study
1. People with MS should continue to advise on the trial throughout
2. Lack of personal experience of CIRCuiTS™ might limit advisors’ ability to offer in-depth recommendations
3. Efforts must be made to recruit diverse people for PPI, as the people most ready to volunteer for PPI roles may not fully represent the trial’s target population in terms of socio-demographics, personality and attitudes to research
Participant recruitment
4. An urban NHS MS service is the best recruitment avenue for reaching a diverse and representative population, providing access to more isolated and less proactive individuals than MS Therapy Centres and charities
5. Healthcare professionals, such as neurologists or MS nurses, should be made familiar with the benefits of cognitive remediation so that they are motivated to aid recruitment
6. Paperwork burden on participants should be minimised
Communicating with potential participants
7. CIRCuiTS™ has many potential benefits for people with MS, such as conferring autonomy and stimulation
8. Certain aspects of the therapy are prone to misunderstanding by lay people unless described clearly
9. Some communicative approaches, such as oversimplifying cognitive difficulties or drawing parallels with mental health conditions, are alienating
Intervention design
10. CIRCuiTS™’s features of tailored goals, personalised difficulty progression, and real-life tasks are well suited to people with MS
11. All cognitive task categories will benefit some individuals with MS
12. Certain tasks, such as those with time pressure, can be particularly challenging but should be included to help build real-life coping strategies
13. Tasks requiring fine motor control or rapid mouse use should be omitted for people with limited hand dexterity
Intervention delivery
14. A schedule of two 1-h therapy sessions per week plus 1 h of independent tasks would be manageable, though it might be challenging for those in employment
15. In-person visits may be preferred by those who are not digitally confident, but travelling to a clinic may impose significant logistical and time burdens, particularly for those in employment
16. Remote sessions will be desirable, easier to schedule, and accessible for most, particularly if help with technical setup is offered
17. At-home therapy visits are unlikely to be sustainable
18. Therapists should have MS-specific training giving them insight into how diverse lived experience is in MS, the cognitive and physical challenges people with MS face, and how factors like mood and fatigue affect cognitive performance
Potential outcomes
19. CIRCuiTS™ has the potential to help people with MS with goal attainment, mood, cognition, fatigue management and carrying out everyday activities
Data collection
20. Hand function, fatigue, temperature sensitivity and mobility could impact the ability of people with MS to engage with CIRCuiTS™ and therefore need to be measured
Ethical considerations
21. People with MS can experience anxiety, denial, frustration and shock at their own cognitive changes
22. The emotional impact of CIRCuiTS™ sessions could exacerbate physical symptoms
23. Hour-long sessions may be difficult when experiencing MS-related fatigue and bladder symptoms unless breaks are offered
24. Transparency about data privacy is crucial

*CIRCuiTS™* Computerised Interactive Remediation of Cognition and Thinking Skills

### Feasibility study protocol

This protocol has been written according to the SPIRIT 2025 statement [36], with recommended adjustments for feasibility trials [37] (see Table S2 in Additional File 1).

#### Aim and design

This trial will determine whether delivering CIRCuiTS™ cognitive remediation therapy to people with MS experiencing cognitive difficulties is feasible and acceptable.

**Fig. 1:**
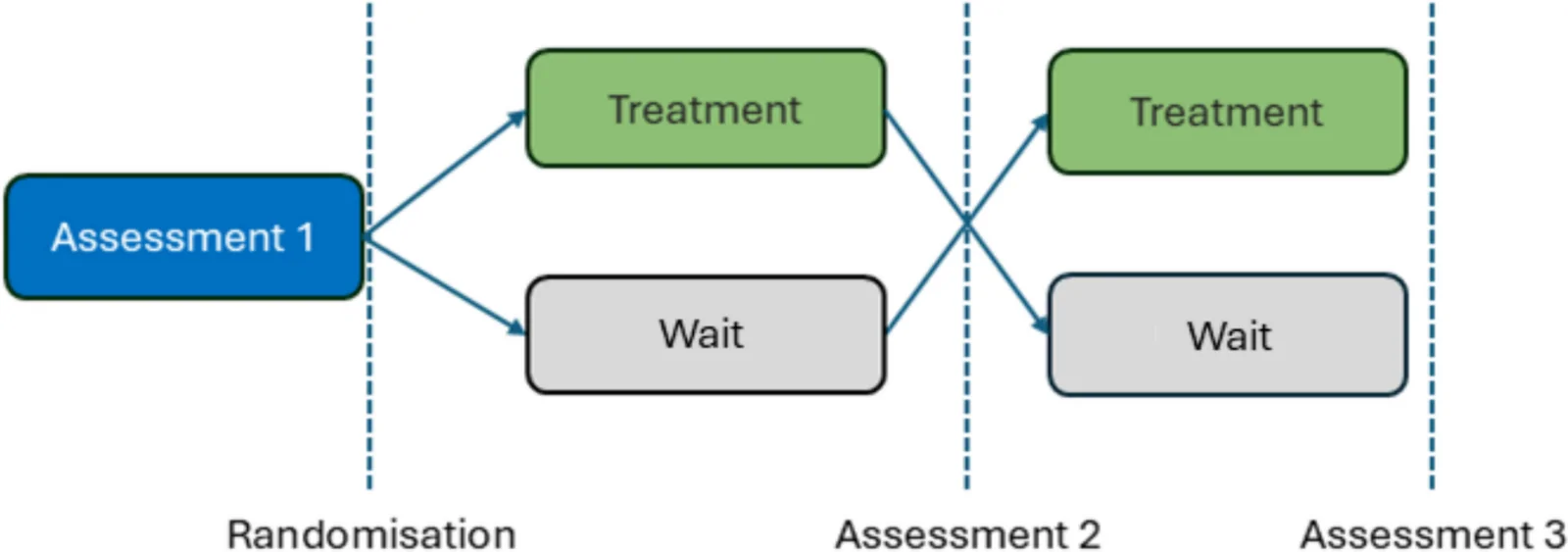
Parallel-group, waitlist-controlled study design

The design is a randomised, parallel-group, waitlist-controlled feasibility trial (Fig. 1). Participants will be randomly assigned, with equal probability, to receive 12 weeks of cognitive remediation either immediately (immediate treatment group) or after 13 weeks’ wait (delayed treatment group). This design offers fair access for all participants to a treatment that has been shown to be effective in other conditions and is anticipated to offer numerous benefits to people with MS (Recommendation 19). The waiting time for the delayed treatment was also acceptable and likely to encourage engagement throughout study assessments (PPI lead design input). At the end of the trial (week 27) there will also be an exit interview covering topics like acceptability of the trial procedures and the treatment provided. As well as the primary outcomes of feasibility and acceptability, measures to be used in a further trial including recovery measures and cognition will be assessed at weeks 0, 14 and 27 (see Table 2). Outcome assessors will be blind to group assignments to reduce measurement bias. In case of unblinding, a different, blinded assessor will complete all further assessments. Each assessment can be split over more than one session in case of fatigue.

**Table 2:**
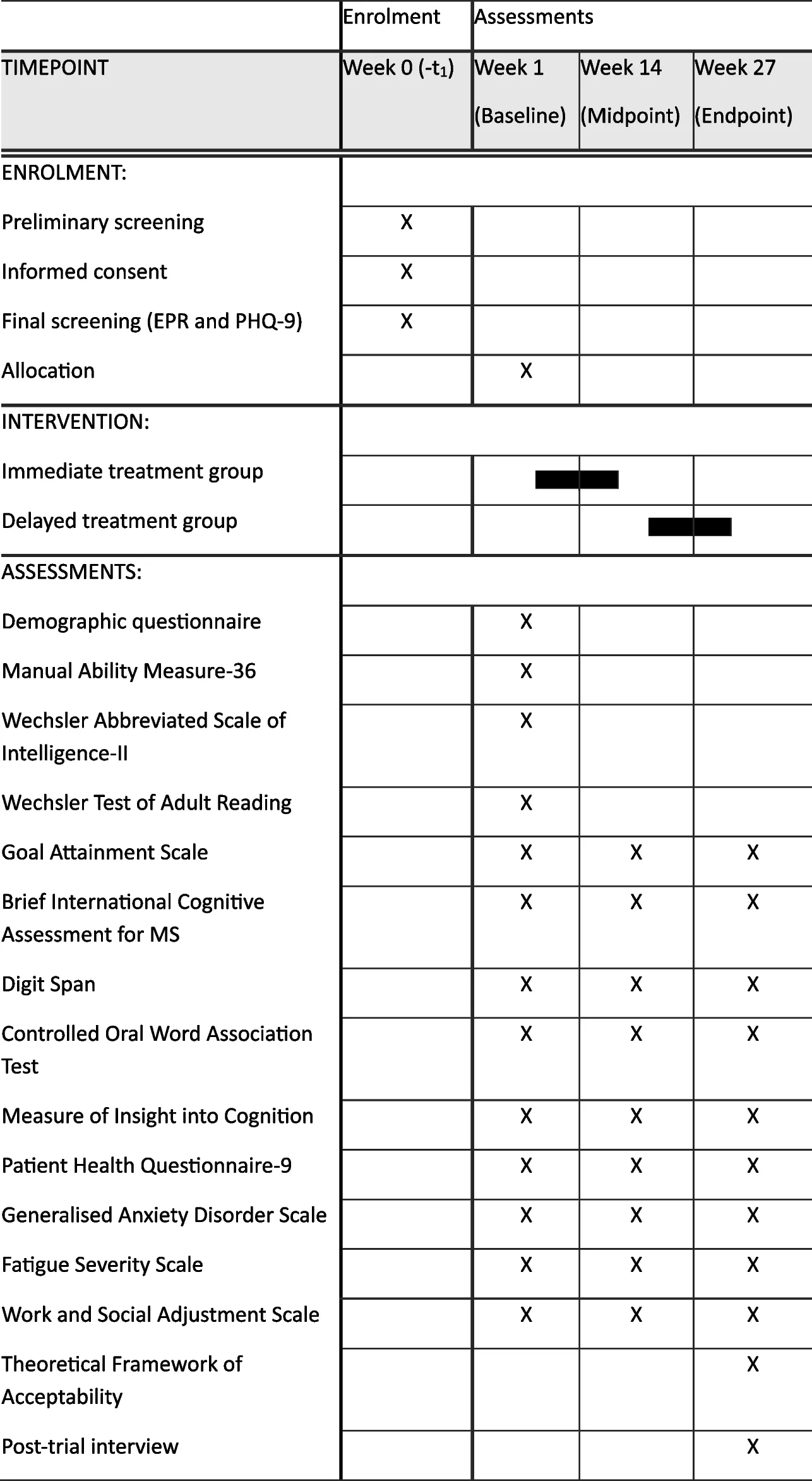
Schedule of enrolment, interventions, and assessments

*EPR* electronic patient records

Randomisation will be performed independently using a computerised randomisation system developed and maintained by the King’s Clinical Trials Unit. It will randomise each individual and use block randomisation with randomly varying block sizes. Randomisation will be initiated by the trial manager via a web interface. Access to the resulting group assignment will be restricted to designated non-blinded team members through a permissions-based system.

The study protocol is registered on ClinicalTrials.gov (NCT06877273) and has ethical approval from the London – Brent Research Ethics Committee (25/LO/0340).

#### Setting and eligibility

To maximise diversity, participants will be recruited from outpatient neurology clinics, including inner-city clinics that have referrals from different ethnic, cultural and socioeconomic groups. This approach increases demographic and clinical diversity and avoids the potential bias introduced by recruiting from charities or MS Therapy Centres, which typically attract more proactive individuals (Recommendation 4).

Eligible participants must be 18 years old or older, have a neurologist-confirmed MS diagnosis and be clinically stable (i.e. not experiencing an acute health crisis that might impact engagement). This ensures suitability for the therapy, and to improve adherence, they must have subjective cognitive challenges related to MS that they would like help with. They must be able to provide consent, have sufficient cognitive and communication abilities to engage in English-language therapy sessions, and be willing and able to attend all sessions and complete homework over 12 weeks. Exclusion criteria are self- or clinician-reported cognitive or physical impairments that would prevent meaningful participation, moderate–severe depression or suicidality as measured with the Patient Health Questionnaire-9 (PHQ-9 [38]; total score > 9, or scoring > 0 on the self-harm item), clinician diagnosis with a psychotic or bipolar disorder, or currently receiving cognitive rehabilitation. Moderate–severe depression is excluded for ethical and methodological reasons: confronting cognitive difficulties during the intervention may have an emotional impact (Recommendation 21), and depression is associated with cognitive functioning [21] and can be improved by cognitive remediation [22], potentially confounding the interpretation of cognitive outcomes.

#### Recruitment procedures

Participants will be recruited through referral from healthcare staff, who will be fully briefed on the potential benefits of cognitive remediation to encourage staff knowledge and engagement to reduce the barriers to recruitment (Recommendations 4 and 5). In addition, we will use our Advisory Group to suggest processes to increase participant recruitment. Potential participants will provide digital written consent and be screened for eligibility. If eligible, they will complete a baseline assessment and be randomised (see above). In line with PPI recommendations, participants will receive £40, optionally as shopping vouchers (Recommendation 6), for each of three assessment meetings and will have travel costs reimbursed (Recommendation 15). There is no reimbursement for attending therapy sessions.

To support informed consent, participant information documents have been optimised to highlight the benefits of the intervention (Recommendation 7) and to describe CIRCuiTS™ without reference to other patient groups (Recommendation 9). These documents, together with the consent form, were reviewed by people with lived experience of mental health problems (Maudsley Biomedical Research Centre’s FAST-R service [39]) at stage 3 of the co-development process to ensure they are easy to understand by all potential participants (Recommendation 8). Typographical and formatting changes were made in response to this review. Family members or caregivers may assist with communication during the consent process to ensure full understanding (Recommendation 24).

#### Intervention: MS-adapted CIRCuiTS™

Workshop discussions confirmed CIRCuiTS™ to be an intervention that aligns well with the needs and priorities of people with MS (Recommendation 10). CIRCuiTS™ is therapist-guided web-based computerised CR software that combines repeated cognitive exercises, strategy teaching and metacognitive skill support linked to personalised functioning goals. Task difficulty is adaptive to the client’s abilities, and the therapist can tailor tasks to address the client’s ability and goals. Its core purpose is to help the client transfer newly learned cognitive skills into real-life functioning (see Reeder and Wykes [40] for a full description of the programme). Based on delivery protocols in a recent large-scale CIRCuiTS™ trial [25] and PPI participants’ endorsement (Recommendation 14), trial participants will be asked to attend two 1-h therapy sessions and complete 1 h of independent tasks per week over 12 weeks, totalling 36 h. All elements of the standard CIRCuiTS™ programme [40] will be provided (Recommendations 11 and 12), though participants with reduced ability to operate a mouse quickly or dexterously will be offered an adapted version omitting tasks that require these abilities (Recommendation 13). Dexterity will be assessed based on the participant’s score on the Manual Ability Measure-36 (MAM-36) [41] and observations of their hand function during the CIRCuiTS™ ‘How to use a mouse’ tutorial, which involves accurate clicking and dragging.

Adaptations have been made to the delivery protocol to promote adherence. Remote sessions will be offered to reduce travel time, enable scheduling flexibility, and reduce discomfort and fatigue, particularly for those with reduced mobility, with in-person sessions as an option (Recommendations 15–17). For those opting for remote sessions, an initial home visit will be offered to establish rapport and resolve technical issues (Recommendation 16). In-person sessions will be located at the participant’s preferred clinic location (Recommendation 15). Remote therapy sessions will be conducted via Microsoft Teams. Breaks will be incorporated into therapy to reduce physical burdens (Recommendation 23).

Therapists will receive standard CIRCuiTS™ training [42] with additional MS-specific training (Recommendation 18) reflecting PPI priorities such as highlighting diverse symptom profiles in MS, the emotional impact of cognitive changes in MS, factors affecting cognitive performance in MS, the potential impacts of physical symptoms on therapy engagement, strategies commonly used to manage cognitive difficulties in MS, and how to communicate about CIRCuiTS™ with people with MS. Adherence to the therapy model, manual and procedures will be monitored in weekly supervision sessions offered by an experienced clinical psychologist.

#### Data collection

*Contextual information* will be collected at baseline on factors that might moderate feasibility or benefits. This will include demographic information (self-report) and clinical data from the participant’s electronic patient record, including their score on the Expanded Disability Status Scale [43] (Recommendation 20), which is assessed by a neurologist at each clinic visit (at least annually in stable disease and more frequently where clinically indicated). For participants recruited at a neurologist appointment, this will have been performed at that appointment. As hand function can be compromised in MS [44] and could affect CIRCuiTS™ MS’ feasibility and acceptability (Recommendation 20), the MAM-36 will also be administered. This is a self-report measure of ability to perform 36 one- or two-handed tasks, with higher total scores indicating better function.

IQ will be assessed with vocabulary and matrix reasoning tests from the Wechsler Abbreviated Scale of Intelligence, Second Edition (WASI-II) [45] and premorbid IQ with the Wechsler Test of Adult Reading (WTAR) [46].

Therapists will record the current temperature at the participant’s location at each session according to data issued by the Met Office (https://www.metoffice.gov.uk/) (Recommendation 20).

Primary outcome: *Trial feasibility and acceptability* will be evaluated quantitatively using Go/No-Go thresholds (Table 3), including self-reported acceptability on the Theoretical Framework of Acceptability (TFA) questionnaire [47], a measure of healthcare intervention acceptability administered at Assessment 3. Acceptability and perceived burden will be further explored through qualitative analysis of semi-structured one-on-one interviews with a trained researcher at Assessment 3.

**Table 3:** Feasibility outcome measures and decision rules

Measure	Operationalisation	‘Go’ criterion	‘No-Go’ criterion
Reach			
Recruitment	% planned sample size recruited	Enrolling ≥ 80% of the planned sample size	
Ethnic enrolment diversity	% sample belonging to most common ethnicity	< 75% participants of a single ethnicity^1^	
Gender enrolment diversity	% participants identifying as male and female	< 50% difference between those identifying as men and women^2^	
Retention			
Completion rate	% completing 27-week outcomes	≥ 50% participants finally retained at 27 weeks	
Midpoint retention	% completing 14-week outcomes	≥ 70% participants retained 14 weeks	
Dropout	% enrolled participants who drop out	< 10% dropout due to adverse events directly related to the intervention	
Safety			
Serious adverse events	Number of serious adverse events (SAEs) reported	No SAEs directly related to the intervention reported	An SAE directly related to the intervention
Manageability of adverse events	Number of adverse events that cause the study to be halted reported	Only mild to moderately severe adverse events that are managed without halting the study	Any adverse event that poses a significant risk to participant safety and cannot be mitigated
Data Integrity			
Data completeness	Overall percentage outcome data complete	≥ 50% completeness in outcome data^3^	
Specific measure completeness	Percentage outcome data complete per measure	≥ 50% complete indicates that measure is feasible	
Operational feasibility	Number of significant operational issues that cannot be resolved	Only resolvable operational issues arise	Significant, unresolvable operational issues arise, e.g. persistent software problems or insufficient staff for the study
Intervention feasibility			
Adherence:	% attending therapy sessions	> 50% participants adherent	
Independent sessions	% attending independent sessions	> 50% participants adherent	
Measure acceptability	% who complete demographic questions	Questions for which ≤ 50% select ‘Prefer not to say’ will be considered feasible	Questions for which > 50% select ‘Prefer not to say’ will not be considered feasible
Acceptability	Mean score on the general acceptability item of the Theoretical Framework of Acceptability [47]	Mean score of > 2.5	

^1^Based on a UK average of 82% White but larger non-White representation in South London

^2^Based on a 1:3 ratio in UK population with MS

^3^Measured as the percentage of measures intended to be collected that were successfully collected across participants

A decision to progress to a larger trial will be based primarily on feasibility of recruitment; if recruitment is not feasible, a definitive trial would not be possible. If this Go criterion is met, decisions regarding progression and the need for substantial amendments to the study design will be informed by the remaining Go/No-Go criteria and findings from the exit interviews. Measures that fail to meet criteria for completeness and acceptability will be omitted or substituted in any future trial.

Secondary outcomes: Changes following treatment will be measured with the following instruments.

1. *Personal recovery* will be assessed with the Goal Attainment Scale (GAS) [48], as it is a personalised measure but scores are on a similar scale, so achievement of goals can be assessed across the group. As individuals with MS will have different types of goals, this was thought to capture that variability and be preferable to participants (Recommendation 19).
2. *Cognitive function* will be assessed (Recommendation 19) through tests of attention, processing speed, working memory, and executive function, following prior work on cognitive vulnerabilities in MS [49–51]. These cognitive domains will be measured on three tests incorporated into the Brief International Cognitive Assessment for Multiple Sclerosis (BICAMS) [50]: (i) Symbol Digit Modalities Test (SDMT) [52], a measure of processing speed and attention, (ii) the Brief Visuospatial Memory Test-Revised (BVMT-R) [53] for visuospatial working memory capacity, and (iii) the California Verbal Learning Test-Second Edition (CVLT-II) [54], for verbal working memory capacity. The BVMT-R was selected for this study for its brevity, reliability, and sensitivity to cognitive changes associated with MS. The oral version of the SDMT will be used to reduce motor demands [55] (Recommendation 20). As this battery does not test executive function, it will be supplemented by the Digit Span test from the WAIS-IV [56] and the Controlled Oral Word Association Test (COWAT) [57]. The participant must perform operations in working memory for the backwards and sequential parts of the Digit Span and apply cognitive flexibility and inhibitory control in the COWAT, all of which are aspects of executive function. Both measures show good test–retest reliability and convergent validity. In addition to these formal tests, we also want to assess another aspect of perceived benefits, the awareness of experiencing cognitive problems, as this may affect the adherence to therapy. This will be assessed using the Measure of Insight into Cognition (MIC) [58].
3. *Mood* is important in MS, and there was some concern that mental health issues may emerge as the participant becomes more aware of their cognitive difficulties (Recommendation 21). Although the therapy and therapist emphasise building on strengths, the trial will exclude potential participants who have moderate to severe depression in case of potential exacerbation during therapy. Mood will be assessed using the PHQ-9 [38], which is a widely used short, self-administered tool that has been validated as a screening tool in MS [59]. Those exceeding a score of 9 (moderate to severe depression) will be excluded from the study. Anxiety will also be measured, as it may affect adherence to the treatment or trial protocol. It will be assessed on the short, self-report Generalised Anxiety Disorder Scale (GAD-7) [60], a tool validated in MS [61] where a higher total score indicates greater anxiety severity.
4. *Fatigue* will be assessed (Recommendation 19) with the Fatigue Severity Scale (FSS) [62], a common measure of the impact of fatigue on daily life that has been validated in MS (e.g. [63, 64]). The total score is the average of the item scores, with higher scores indicating greater severity and impact.
5. *General ability to carry out everyday activities* will be assessed (Recommendation 19) using the Work and Social Adjustment Scale (WSAS) [65], comprising questions about how the participant’s condition, in this case MS, affects their daily life.

#### Data management

Any personally identifying information will be stored on a secure server separately from non-identifying information and password protected, with access rights minimised to research team members who need to view it to carry out trial activities. Paper copies will be stored in a locked cabinet.

All assessment data will be stored using a unique numeric participant identifier separately from all personally identifiable information. Standardised test data will be recorded using publisher-provided scoring forms. Self-report measures and summary statistics from standardised tests will be entered by the assessor into the Qualtrics platform. Qualtrics range validation checks will be used to promote data quality. Data verification will be conducted at the midpoint and end of the study by an independent scorer, who will calculate summary statistics for a random 10% sample.

Identifiable data will be stored for 24 months, after which it will be converted to a de-identified format and retained for a further 5 years.

#### Analysis

This analysis plan was developed in collaboration with a statistician at King’s College London.

##### Aim 1: Feasibility and acceptability

Feasibility and quantitative aspects of acceptability will be measured quantitatively through evaluating trial performance against the Go/No-Go criteria (Table 3).

Acceptability will be explored further through framework analysis of qualitative data [66] from participant interviews using NVivo version 14 [35]. A researcher will check the transcriptions then review and index the data. A thematic framework followed by a summary in a framework matrix will be created by two independent researchers. Following good research practice [27] and a commitment to PPI, participants will be asked to provide feedback on the results of this analysis as part of member checking, which will be synthesised into reports disseminating the research.

##### Aim 2: Potential benefits

Quantitative outcome data will be analysed on an intention-to-treat basis, with all randomised participants included regardless of adherence. The focus will be on descriptive statistics and 95% confidence intervals to assess the likely range of treatment effects. The GAS T-score will be evaluated using a linear mixed model.

Means and standard deviations for scores on measures of the secondary outcomes will be calculated at each assessment point for both groups and for groupwise within-subject change in score across assessment points, and the distribution of scores will be summarised to assess normality.

##### Sample size

Twenty-four participants will be recruited. Drawing on prior experience, this number is considered sufficient to estimate key feasibility outcomes (e.g. recruitment and dropout rates), meaningfully assess the practical challenges that might arise from participation (Recommendation 20), and recruit a range of participants with different characteristics. It also follows recommendations to include at least 12 participants per pilot group (24 in total) to adequately estimate the variability of outcome parameters in a larger definitive trial [67]. This target sample size was also considered feasible by the recruiting site within the planned recruitment period.

#### Data and trial monitoring

Data and trial monitoring will be carried out by a Steering Committee that includes three academic clinical psychology trialists, a mental health professional, two neurologists and two people with MS. This committee will regularly review study methodology, management, and analysis to ensure the study stays patient-focused and complies with ethical trial procedures. The Chief Investigator oversees the regular monitoring of protocol adherence, consenting procedures, and data quality. Any concerns will be reported to the sponsor.

#### Potential adverse events

Being assessed for and discussing cognitive difficulties may trigger emotional distress and subsequent exacerbation of physical symptoms, as described by PPI advisors (Recommendations 21 and 22). The research team will therefore monitor participants' mental health throughout the study using observation and the PHQ-9 to identify signs of depression or distress, referring participants to mental health support services if needed. All adverse events (Table S3 in Additional File 1) will be tracked, considered by a clinical expert for their validity and severity. If it is an adverse event, it is reported to the Steering Committee and addressed, with participants being given the option to pause or withdraw from the study. Serious adverse events will be reported immediately and managed according to established safety protocols and reported to the Ethics Committee.

#### Ethics and dissemination

##### Protocol amendments

If the study requires substantial amendments, approval will be sought from the sponsors and research ethics committee, and the changes will be registered with ClinicalTrials.gov. If amendments concern or affect participants in any way, they will be informed about the changes, and additional consent will be requested if necessary.

##### Confidentiality

To ensure the protection of personal data in compliance with regulations such as GDPR, patient data will be de-identified to safeguard identities and access to personal data will be controlled through role-based permissions and multi-factor authentication (MFA). Microsoft Teams will be used to audio record interviews during encrypted sessions, and these recordings will be destroyed after transcription.

Clear, accessible information on data privacy arrangements has been included in all patient information documents, and trial researchers will provide further information to participants at their request.

##### Dissemination

Results will be reported in peer-reviewed scientific journals, internal reports, and conference presentations, with an opportunity for PPI advisors to be involved. We will also enlist the Advisory group and MS Advocacy groups in dissemination activities, including blogs and podcasts accessible to a lay audience. Participants will receive an accessible summary of the study’s findings after the analysis is complete.

##### Involvement of Lived Experience in the study

Following the latest guidelines from the CONSORT group [30], we have already involved people with lived experience in three ways. First with a lived experience co-applicant, second through protocol co-development in a 2-day PPI workshop and finally through our review of study documents.

Based on Recommendation 1, we have increased involvement on the Steering Group with the addition of a further person with lived experience of MS and set up a separate dedicated MS-PPI Advisory group that will be consulted throughout on recruitment, general study management, data collection, analysis and the write-up of the results. We emphasise diversity in advisor recruitment (Recommendation 3) and will include participation in a CIRCuiTS™ session to increase familiarity with the programme (Recommendation 2). We hope that some Advisory group members will agree to be authors of the final publication and other dissemination activities.

## Discussion

This protocol demonstrates how patient and public involvement (PPI) can meaningfully inform the development of a clinical trial designed to evaluate the feasibility, acceptability and potential benefit of delivering CIRCuiTS™ cognitive remediation therapy to people with MS. The proposed trial addresses an important unmet need—to develop interventions that improve the everyday functioning of people living with MS-related cognitive difficulties. Through sustained collaboration, PPI advisors made substantial contributions to both protocol development and therapy provision design, increasing the likelihood that the trial procedures will be feasible and acceptable to participants, consistent with findings from previous research [29, 68].

A key strength of this work is the transparent and explicit reporting of PPI contributions to trial design. While the value of meaningful PPI is increasingly recognised by researchers, funders and journals, e.g. [69–71], its impact is often not reported or reported in a tokenistic manner [72]. By clearly documenting where and how PPI influenced this protocol, this paper provides a practical model for integrating and reporting PPI within trial development, supporting greater accountability and reproducibility in this area.

The trial also has notable methodological strengths. It has a parallel-group, waitlist-controlled design, which maximises data collection from a small sample, provides a control group, and ensures all participants receive the intervention, potentially enhancing recruitment. Additionally, the enthusiasm of the PPI advisors about its potential benefits suggests the intervention may be well-received by people with MS experiencing cognitive difficulties.

## Supplementary Information


Supplementary Material 1. Table S1. Thematic framework developed through analysis of data from patient and public involvement (PPI) workshops. Table S2. SPIRIT 2025 checklist of items to address in a randomised trial protocol. Table S3. Potential adverse events consistent with available information about CIRCuiTS™.

## Data Availability

The datasets used and/or analysed during the current study are available from the trial research team on reasonable request.

## Abbreviations

CIRCuiTS™: Computerised Interactive Remediation of Cognition and Thinking Skills
CONSORT: CONsolidated Standards Of Reporting Trials
CRT: Cognitive remediation therapy
EPR: Electronic patient record
MAM-36: Manual Ability Measure-36
MS: Multiple sclerosis
PHQ-9: Patient Health Questionnaire-9
SPIRIT: Standard Protocol Items: Recommendations for Interventional Trials
