# Supplementary material for: Co-development of a protocol for a randomised controlled feasibility trial of CIRCuiTS™ cognitive remediation therapy for people with multiple sclerosis
Source: Pilot Feasibility Stud. 2026 Jun 20;12:128. doi: 10.1186/s40814-026-01862-2 (PMC13527947; doi:10.1186/s40814-026-01862-2)
Supplement: Supplementary file 1 [file 40814_2026_1862_MOESM1_ESM.pdf]

Co-development of a protocol for a randomised controlled feasibility trial of CIRCuiTS™ cognitive remediation therapy for people with multiple sclerosis

Additional file 1

Contents

Table S1. Thematic framework developed through analysis of data from patient and public involvement (PPI) workshops

Table S2. SPIRIT 2025 checklist of items to address in a randomized trial protocol

Table S3. Potential adverse events consistent with available information about CIRCuiTS™

Table S1. Thematic framework developed through analysis of data from patient and public involvement (PPI) workshops

1. **PPI**
    - 1.1 Limitations
        - 1.1.1 Can't know utility without using CIRCuiTS
        - 1.1.2 Non-representativeness
    - 1.2 Benefits
        - 1.2.1 Expertise beyond MS

2. **Recruitment strategy**
    - 2.1 Recruitment avenue
        - 2.1.1 MS Centres
        - 2.1.2 NHS MS clinics
        - 2.1.3 Special interest groups
    - 2.2 Types of diversity to capture
        - 2.2.1 Education, ethnicity and SES
        - 2.2.2 Neurodevelopmental differences
        - 2.2.3 Personal characteristics
        - 2.2.4 Access to stimulation
    - 2.3 Barriers to recruitment
        - 2.3.1 Gatekeepers
        - 2.3.2 Participants
    - 2.4 Facilitators of recruitment
        - 2.4.1 Gatekeepers with frequent patient contact

2.4.2 Persuade specific gatekeepers

2.5 Content for communications

2.5.1 Anticipated benefits of CIRCuiTS

2.5.3 Turn offs

2.5.4 Descriptions of how intervention works

2.5.5 Points requiring clarification

---

3. **Intervention content**

3.1 Endorsement of existing elements

3.1.1 Tailored goals

3.1.2 Tailored speed of difficulty increase

3.1.3 Tasks relevant to daily life

3.1.4 Value of early success

3.1.5 Initial assessment

3.2 Value of including challenging tasks

3.3 Value of covering a range of cognitive functions

3.4 Wished-for content

3.4.1 Tailored starting difficulty

3.4.2 Tailored to motor skills

3.4.3 Pause logging

---

4. **Intervention delivery**

4.1 Barriers to engagement

4.1.1 Fatigue

4.1.2 Language barrier

4.1.3 Temperature sensitivity

4.1.4 Therapist quality

4.2 Facilitators of engagement

4.2.1 Assurance of therapist quality

4.2.2 Finite course of therapy

4.3 Acceptability of homework component

4.4 Acceptability of a blended approach

4.4.1 Negative

4.4.2 Positive

4.5 Acceptability of proposed scheduling

4.5.1 One-hour sessions

4.5.2 Two sessions per week

4.6 Acceptability of therapy venues

4.6.1 Home visit

4.6.2 In-clinic sessions

4.6.3 Remote sessions

5. **Therapist recruitment and training content**

5.1 Therapist recruitment

5.1.1 Desired characteristics

5.1.2 Perspectives on hiring a single therapist

5.2 Nature of cognitive change in MS

5.2.1 Cognitive difficulties

5.2.2 Commonality with other conditions

5.2.3 Complex

5.2.4 Progressive

5.2.5 Sensitive topic

5.2.6 Under-recognised by others

5.2.7 Unique

5.3 Impactors of cognitive performance in MS

5.3.1 Person factors

5.3.2 Task factors

5.3.3 Environmental factors

5.4 Emotional reactions to cognitive changes in MS

5.4.1 Shock and fear

5.4.2 Denial

5.4.3 Frustration

5.5 Physical symptoms that may impact therapy

5.5.1 Fatigue

5.5.2 Hand function

5.5.3 Temperature sensitivity

5.5.4 Emotional escalation of physical problems

5.5.5 Incontinence

5.6 Diversity in MS

5.6.1 Diversity of lived experience

5.6.2 Diversity in cognitive ability

5.6.3 Diversity in awareness regarding cognitive change

5.7 Strategies already used

5.7.1 Acceptance

5.7.2 Mask problems from others

5.7.3 Medication

5.7.4 Memory aids

5.7.5 Resting

5.7.6 Self-monitoring

5.8 Appropriate communication strategies

---

6. **Data collection**

6.1 Physical symptoms that could impact CIRCuiTS feasibility in MS

6.2 Impactors of cognitive performance in MS

6.3 Emotional reactions to cognitive changes in MS

---

7. **Ethical considerations**

7.1 Potential emotional harms from CIRCuiTS

7.1.1 Emotional effects of cognitive testing

7.1.2 Emotions triggered by awareness of cognitive changes

7.2 Potential physical harms from CIRCuiTS

7.2.1 Fatigue

7.2.2 Emotional escalation of physical problems

7.3 Potential harms from therapy venue

7.3.1 Home visit

7.3.2 In-clinic

7.3.3 Remote on computer

7.4 Data privacy

7.4.1 Concerns

7.4.2 Value of transparency

---

8. **Sustainability**
8.1 Potential for implementation in the NHS
8.1.1 Barriers
8.1.2 Facilitators
8.1.3 Ideas for later expansion of reach
8.2 Acknowledgement that trial and real-world requirements differ

Table S2. SPIRIT 2025 checklist of items to address in a randomized trial protocol*

Section / Topic	No	SPIRIT 2025 checklist item description	Reported on page no.
**Administrative information**			
Title	1a	Title stating the trial design, population, and interventions, with identification as a protocol	1
	1b	Structured summary of trial design and methods, including items from the World Health Organization Trial Registration Data Set	NA
Protocol version	2	Version date and identifier	19
Roles and responsibilities	3a	Names, affiliations, and roles of protocol contributors	1
	3b	Name and contact information for the trial sponsor	20
	3c	Role of trial sponsor and funders in design, conduct, analysis, and reporting of trial; including any authority over these activities	20
	3d	Composition, roles, and responsibilities of the coordinating site, steering committee, endpoint adjudication committee, data management team, and other individuals or groups overseeing the trial, if applicable	17
**Open science**			
Trial registration	4	Name of trial registry, identifying number (with URL), and date of registration. If not yet registered, name of intended registry	2
Protocol and statistical analysis plan	5	Where the trial protocol and statistical analysis plan can be accessed	NA
Data sharing	6	Where and how the individual de-identified participant data (including data dictionary), statistical code, and any other materials will be accessible	19
Funding and conflicts of interest	7a	Sources of funding and other support (e.g., supply of drugs)	20
	7b	Financial and other conflicts of interest for principal investigators and steering committee members	20
Dissemination policy	8	Plans to communicate trial results to participants, healthcare professionals, the public, and other relevant groups (e.g., reporting in trial registry, plain language summary, publication)	18
**Introduction**			
Background and rationale	9a	Scientific background and rationale for future definitive trial, including summary of relevant studies (published and unpublished) examining benefits and harms for each intervention	3
	9b	Explanation for choice of comparator	7
	9c	Reasons for randomised pilot trial (CONSORT extension item 2a*)	3
	9d	Specific objectives or research questions for pilot trial (CONSORT extension item 2b*)	4
Objectives	10	Specific objectives related to benefits and harms	4

Methods: Patient and public involvement, trial design			
Patient and public involvement	11	Details of, or plans for, patient or public involvement in the design, conduct, and reporting of the trial	4-7, 18
Trial design	12	Description of trial design including type of trial (e.g., parallel group, crossover), allocation ratio, and framework (e.g., superiority, equivalence, non-inferiority, exploratory)	7
**Methods: Participants, interventions, and outcomes**			
Trial setting	13	Settings (e.g., community, hospital) and locations (e.g., countries, sites) where the trial will be conducted	9
Eligibility criteria	14a	Eligibility criteria for participants	10
	14b	If applicable, eligibility criteria for sites and for individuals who will deliver the interventions (e.g., surgeons, physiotherapists)	NA
Intervention and comparator	15a	Intervention and comparator with sufficient details to allow replication including how, when, and by whom they will be administered. If relevant, where additional materials describing the intervention and comparator (e.g., intervention manual) can be accessed	10-11
	15b	Criteria for discontinuing or modifying allocated intervention/comparator for a trial participant (e.g., drug dose change in response to harms, participant request, or improving/worsening disease)	17
	15c	Strategies to improve adherence to intervention/comparator protocols, if applicable, and any procedures for monitoring adherence (e.g., drug tablet return, sessions attended)	9,10
	15d	Concomitant care that is permitted or prohibited during the trial	NA
Outcomes	16	Completely defined prespecified assessments or measurements to address each pilot trial objective specified in 2b, including how and when they were assessed (CONSORT feasibility trial extension item 6a)	12-16
Harms	17	How harms are defined and will be assessed (e.g., systematically, non-systematically)	17
Participant timeline	18	Time schedule of enrollment, interventions (including any run-ins and washouts), assessments, and visits for participants. A schematic diagram is highly recommended (see Figure)	8-9
Sample size	19	Rationale for numbers in the pilot trial (CONSORT extension item 7a*)	17
Recruitment	20	Strategies for achieving adequate participant enrollment to reach target sample size	10
**Methods: Assignment of interventions**			
Randomization:			
Sequence generation	21a	Who will generate the random allocation sequence and the method used	8
	21b	Type of randomization (simple or restricted) and details of any factors for stratification. To reduce predictability of a random sequence, other details of any planned restriction (e.g., blocking) should be provided in a separate document that is unavailable to those who enroll participants or assign interventions	8

Allocation concealment mechanism	22	Mechanism used to implement the random allocation sequence (e.g., central computer/telephone; sequentially numbered, opaque, sealed containers), describing any steps to conceal the sequence until interventions are assigned	8
Implementation	23	Whether the personnel who will enroll and those who will assign participants to the interventions will have access to the random allocation sequence	8
Blinding	24a	Who will be blinded after assignment to interventions (e.g., participants, care providers, outcome assessors, data analysts)	8
	24b	If blinded, how blinding will be achieved and description of the similarity of interventions	8
	24c	If blinded, circumstances under which unblinding is permissible, and procedure for revealing a participant's allocated intervention during the trial	8
**Methods: Data collection, management, and analysis**			
Data collection methods	25a	Plans for assessment and collection of trial data, including any related processes to promote data quality (e.g., duplicate measurements, training of assessors) and a description of trial instruments (e.g., questionnaires, laboratory tests) along with their reliability and validity, if known. Reference to where data collection forms can be accessed, if not in the protocol	15-16
	25b	Plans to promote participant retention and complete follow-up, including list of any outcome data to be collected for participants who discontinue or deviate from intervention protocols	
Data management	26	Plans for data entry, coding, security, and storage, including any related processes to promote data quality (e.g., double data entry; range checks for data values). Reference to where details of data management procedures can be accessed, if not in the protocol	16
Statistical methods	27	Methods used to address each pilot trial objective whether qualitative or quantitative (CONSORT extension item 12*)	16
**Methods: Monitoring**			
Data monitoring committee	28a	Composition of data monitoring committee (DMC); summary of its role and reporting structure; statement of whether it is independent from the sponsor and funder; conflicts of interest and reference to where further details about its charter can be found, if not in the protocol. Alternatively, an explanation of why a DMC is not needed	17
	28b	Explanation of any interim analyses and stopping guidelines, including who will have access to these interim results and make the final decision to terminate the trial	NA
Trial monitoring	29	Frequency and procedures for monitoring trial conduct. If there is no monitoring, give explanation	17
**Ethics**			
Research ethics approval	30	Plans for seeking research ethics committee/institutional review board approval	8
Protocol amendments	31	Plans for communicating important protocol modifications to relevant parties	17
Consent or assent	32a	Who will obtain informed consent or assent from potential trial participants or authorized proxies, and how	10
	32b	Additional consent provisions for collection and use of participant data and biological specimens in ancillary studies, if applicable	NA

Confidentiality	33	How personal information about potential and enrolled participants will be collected, shared, and maintained in order to protect confidentiality before, during, and after the trial	18
Ancillary and post-trial care	34	Provisions, if any, for ancillary and post-trial care, and for compensation to those who suffer harm from trial participation	NA

*This checklist (1) has been adjusted to include items from the CONSORT 2010 extension to randomised pilot and feasibility trials (2), as recommended by the editors of Pilot and Feasibility Studies (3).

References

1. Chan AW, Boutron I, Hopewell S, Moher D, Schulz KF, Collins GS, et al. SPIRIT 2025 statement: updated guideline for protocols of randomised trials. BMJ [Internet]. 2025 Apr 28 [cited 2025 Dec 16];389:e081477. Available from: https://www.bmj.com/lookup/doi/10.1136/bmj-2024-081477

2. Eldridge SM, Chan CL, Campbell MJ, Bond CM, Hopewell S, Thabane L, et al. CONSORT 2010 statement: extension to randomised pilot and feasibility trials. BMJ [Internet]. 2016 Oct 24 [cited 2025 Dec 16];i5239. Available from: https://www.bmj.com/lookup/doi/10.1136/bmj.i5239

3. Thabane L, Lancaster G. A guide to the reporting of protocols of pilot and feasibility trials. Pilot Feasibility Stud [Internet]. 2019 Dec [cited 2025 Dec 16];5(1):37, s40814-019-0423–8. Available from: https://pilotfeasibilitystudies.biomedcentral.com/articles/10.1186/s40814-019-0423-8

© 2025 Chan A-W et al. This is an Open Access article distributed under the terms of the Creative Commons Attribution License (https://creativecommons.org/licenses/by/4.0/), which permits unrestricted use, distribution, and reproduction in any medium, provided the original work is properly cited.

Table S3. Potential adverse events consistent with available information about CIRCuiTS™

Event	Monitored by
Headache	Participant self-report
Mood disturbances	Participant self-report<br>Scores on the Patient Health Questionnaire-9 and Generalized Anxiety Disorder-7 at assessment
Increased fatigue	Participant self-report<br>Therapist observations
